# Cognitive outcomes in patients with essential tremor treated with deep brain stimulation: a systematic review

**DOI:** 10.3389/fnhum.2024.1319520

**Published:** 2024-02-02

**Authors:** Jamal Al Ali, Maureen Lacy, Mahesh Padmanaban, Widad Abou Chaar, Hannah Hagy, Peter C. Warnke, Tao Xie

**Affiliations:** ^1^Department of Neurology, University of Chicago Medicine, Chicago, IL, United States; ^2^Department of Psychiatry and Behavioral Neuroscience, University of Chicago Medicine, Chicago, IL, United States; ^3^Department of Neurological Surgery, University of Chicago Medicine, Chicago, IL, United States

**Keywords:** essential tremor, deep brain stimulation, cognitive function, speech, VIM, cZi, PSA, review

## Abstract

**Introduction:**

Essential tremor (ET) is a common neurological disease. Deep brain stimulation (DBS) to the thalamic ventral intermediate nucleus (VIM) or the adjacent structures, such as caudal zona incerta/ posterior subthalamic area (cZi/PSA), can be effective in treating medication refractory tremor. However, it is not clear whether DBS can cause cognitive changes, in which domain, and to what extent if so.

**Methods:**

We systematically searched PubMed and the Web of Science for available publications reporting on cognitive outcomes in patients with ET who underwent DBS following the PICO (population, intervention, comparators, and outcomes) concept. The PRISMA guideline for systematic reviews was applied.

**Results:**

Twenty relevant articles were finally identified and included for review, thirteen of which were prospective (one also randomized) studies and seven were retrospective. Cognitive outcomes included attention, memory, executive function, language, visuospatial function, and mood-related variables. VIM and cZi/PSA DBS were generally well tolerated, although verbal fluency and language production were affected in some patients. Additionally, left-sided VIM DBS was associated with negative effects on verbal abstraction, word recall, and verbal memory performance in some patients.

**Conclusion:**

Significant cognitive decline after VIM or cZi/PSA DBS in ET patients appears to be rare. Future prospective randomized controlled trials are needed to meticulously study the effect of the location, laterality, and stimulation parameters of the active contacts on cognitive outcomes while considering possible medication change post-DBS, timing, standard neuropsychological battery, practice effects, the timing of assessment, and effect size as potential confounders.

## Introduction

Essential tremor (ET) is one of the most common neurological diseases, with an estimated global prevalence of about 25 million in 2020 (Louis and Ferreira, 2010; Song et al., 2021). Medical treatment, including propranolol, primidone, and topiramate, has been shown to improve tremor severity by approximately 50% (Deuschl et al., 2011; Hopfner and Deuschl, 2020). For patients with medically refractory symptoms, deep brain stimulation (DBS) has been well-accepted as an efficacious treatment alternative with a higher efficacy (Deuschl et al., 2011).

Historically, the ventral intermediate nucleus of the thalamus (VIM) has been the main target of DBS in treating ET (Benabid et al., 1991; Sydow et al., 2003; Wharen et al., 2017). More recently, it has become evident that stimulating the adjacent areas of the VIM, such as the ventral border of the VIM or the ventrolateral or posterior (VL/VLp) thalamus or beneath in an area referred to as the posterior subthalamic area (PSA), which includes the zona incerta (Zi, caudal and rostral, or cZi and rZi) and prelemniscal radiation, is equally effective or could be more efficient with less stimulating energy needed and less stimulating related side effects in treating patients with ET and other tremors (Herzog et al., 2007; Barbe et al., 2011, 2018; Sandvik et al., 2012; Xie et al., 2012; Pedrosa et al., 2014; Ramirez-Zamora et al., 2016; Blomstedt et al., 2018; Al-Fatly et al., 2019). Stimulation within the PSA/cZi is proposed to take advantage of the small anatomical area where a large proportion of cerebellothalamic afferents can be targeted before the fibers spread out to enter the VIM nucleus (Herzog et al., 2007; Xie et al., 2012; Ramirez-Zamora et al., 2016), and also could be due to its proximity to the dentatorubrothalamic tract (Dembek et al., 2020).

DBS is an invasive procedure with multiple risks ranging from stimulation-related side effects to surgical and equipment failure-related complications (Della Flora et al., 2010). Cognitive changes have been reported as a side effect of DBS in some cases, largely depending on the DBS targets and disease mechanism (Cernera et al., 2019). The cognitive side effects of DBS in patients with ET have not been well studied due to the limited cases available, even in the most recent review (Cernera et al., 2019). As new studies and trials are emerging, here we have systematically reviewed the up-to-date literature in search of studies reporting on cognitive outcomes in patients with ET who underwent DBS targeting the VIM and its adjacent structures.

## Methods

**Figure 1:**
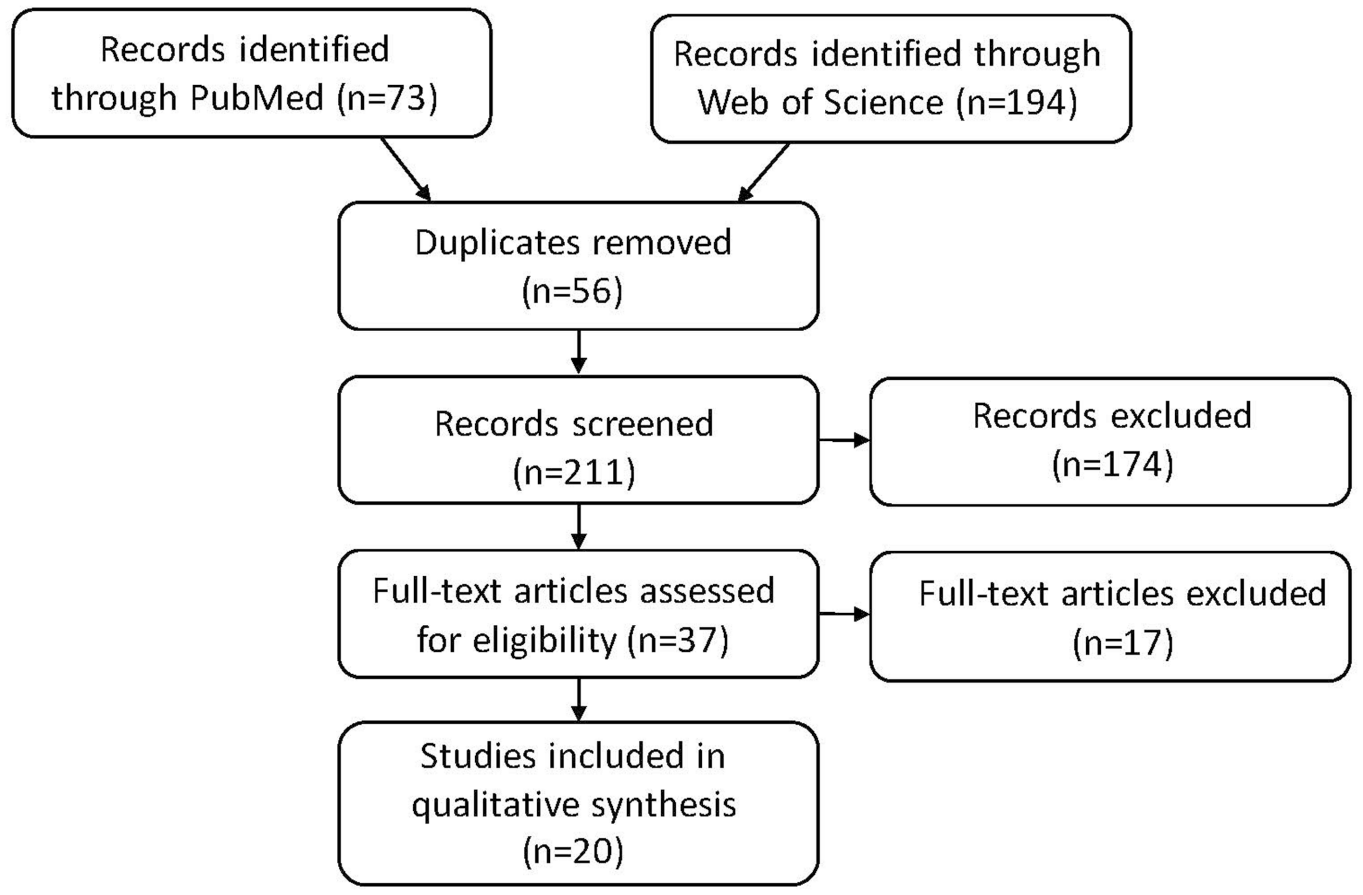
PRISMA flow diagram: literature search and selection with numbers of articles at each stage.

We systematically searched PubMed and the Web of Science in March 2023 for all available publications in English by keywords following PICO concepts: population (patients with essential tremor or ET), intervention (DBS or deep brain stimulation), comparators [DBS targets (VIM, VL/VLp, PSA/cZi), pre-/post-DBS, DBS settings (ON/OFF, location and laterality of the active contact, amplitude of voltage or current, pulse width, and stimulation frequency), medication state (with/without changes after DBS procedure or during the ON/OFF assessment), age at onset of ET, DBS durations at the assessment, other non-ET groups of healthy controls (HCs) or other neurological disorders with DBS as comparisons within the studies mainly for ET, and types of study designs (retrospective vs. prospective and open vs. blind)], and outcomes (neuropsychological outcomes, including mood related variables). We followed the PRISMA guideline for systematic reviews, with the flow chart of the literature search and selection process of the review being depicted in Figure 1. A total of 73 publications were found in PubMed and 194 from Web of Science as of March 2023. After removing the duplicate entries, screening was performed to narrow the publications down to 37, excluding reviews, comments, viewpoints, author responses, letters, book chapters, single case reports with insufficient information, and meeting abstracts. Then the full texts were assessed, and we further removed studies without clear outcome measures on cognitive function. We finally identified 20 relevant articles.

## Results

Each of the twenty articles is listed in detail in Supplementary Table S1, with information on the disease status [e.g., ET, Parkinson’s disease (PD), multiple sclerosis (MS)] and HC, DBS targets, and laterality, basic demographics, study design, DBS settings, medication status (e.g., before and after the DBS device implantation, and/or during the OFF/ON DBS assessment), and neuropsychological findings (including mood related variables). Thirteen articles were prospective design studies (Tröster et al., 1999; Fields et al., 2003; Loher et al., 2003; Woods et al., 2003; Burdick et al., 2011; Fytagoridis et al., 2013; Heber et al., 2013; Pedrosa et al., 2014; Ehlen et al., 2016, 2017; Klein et al., 2017; Philipson et al., 2019; Wang et al., 2020), with one of them a prospective randomized controlled study (Pedrosa et al., 2014). Seven articles utilized a retrospective design (Ehlen et al., 2014; Krugel et al., 2014; Jones et al., 2020; Dhima et al., 2021; Tiedt et al., 2021; Wang et al., 2021; Kielb et al., 2022).

Ten studies reported cognitive outcomes of patients following VIM-DBS for ET (Tröster et al., 1999; Fields et al., 2003; Woods et al., 2003; Ehlen et al., 2016; Klein et al., 2017; Jones et al., 2020; Wang et al., 2020, 2021; Dhima et al., 2021; Kielb et al., 2022), two studies following cZi DBS (Fytagoridis et al., 2013; Philipson et al., 2019), and two following VL/VLp DBS (Heber et al., 2013; Pedrosa et al., 2014). Six studies were compared, four of them compared VIM DBS in patients with ET with STN/GPi-DBS in patients with PD and HCs (Burdick et al., 2011; Ehlen et al., 2014; Krugel et al., 2014; Tiedt et al., 2021), one compared ET patients with VIM DBS to HCs (Ehlen et al., 2017) and one compared VIM DBS to treat tremors in patients with ET, PD, and MS (Loher et al., 2003). Thirteen studies compared cognitive outcomes pre- to post-DBS (Tröster et al., 1999; Fields et al., 2003; Woods et al., 2003; Burdick et al., 2011; Fytagoridis et al., 2013; Heber et al., 2013; Klein et al., 2017; Philipson et al., 2019; Jones et al., 2020; Wang et al., 2020, 2021; Dhima et al., 2021; Kielb et al., 2022), and nine compared cognition at DBS ON to OFF status (Loher et al., 2003; Fytagoridis et al., 2013; Heber et al., 2013; Ehlen et al., 2014, 2016, 2017; Krugel et al., 2014; Pedrosa et al., 2014; Tiedt et al., 2021). Seven studies included unilateral DBS (Tröster et al., 1999; Fields et al., 2003; Loher et al., 2003; Woods et al., 2003; Burdick et al., 2011; Ehlen et al., 2014; Kielb et al., 2022), five included bilateral DBS (Krugel et al., 2014; Pedrosa et al., 2014; Ehlen et al., 2017; Klein et al., 2017; Wang et al., 2020) and eight included a mixture of patients with both unilateral and bilateral DBS (Fytagoridis et al., 2013; Heber et al., 2013; Ehlen et al., 2016; Philipson et al., 2019; Jones et al., 2020; Dhima et al., 2021; Tiedt et al., 2021; Wang et al., 2021). We summarized the findings from these studies below, with details in Supplementary Table S1.

### VIM (and VL/VLp) DBS

The earliest study on cognitive outcomes in patients with VIM-DBS for ET was a prospective study of 40 patients with unilateral VIM-DBS (Tröster et al., 1999). Using a comprehensive battery of cognitive tests, this study found that 3 months after the VIM DBS, patients demonstrated statistically significant and clinically modest improvement in the cognitive domains of attention, memory, and visuospatial function as compared to 1 month prior to DBS surgery. A follow-up study 12 months post-DBS continued to show statistically significant improvements in a cognitive screening measure and tasks of fine visuomotor coordination, visuoperceptual gestalt formation, and verbal memory (Fields et al., 2003). No group-wise declines in cognition were observed, but more patients showed declines than improvements on language and visual memory tests. There were minimal changes in ET medications at 3- and 12 months post-DBS.

Another early study in 2003 was conducted on 49 patients with unilateral VIM DBS for ET and showed that 55% of patients demonstrated mild cognitive decrement (Woods et al., 2003). It was found that the group of patients with a cognitive decrement had significantly higher pulse width (>120 μs), and were more likely to have undergone left (dominant hemisphere) DBS. This study did not report on the specific cognitive domains that were affected, on the reasons for higher pulse width used, or its relationship to precise electrode location in the VIM, but the authors reported controlling for medication changes. In a study of 9 patients with ET, PD, or MS, left-sided VIM stimulation was associated with impairment in short-delayed word recall (Loher et al., 2003). The effect of laterality was further investigated in a retrospective analysis of 50 ET patients, with 14 of them on bilateral, and 36 on unilateral VIM DBS (Dhima et al., 2021). Individual-level analysis showed that 46% of patients experienced a subtle decline in overall cognition pre- and post-DBS, which correlated with higher right-sided stimulation amplitude, as did worsened visuospatial judgment. On the other hand, the longer left-sided pulse width was correlated with a decline in verbal memory performance, and higher left-sided stimulation frequency was correlated with increased perseveration during novel problem-solving. Notably, in this study, there was no group-level cognitive decline pre- and post-DBS. Additionally, medications were decreased in 46% of patients post-DBS without any benefit on cognitive outcomes in a *post-hoc* analysis.

Following the first study that reported a decline in verbal fluency in ET patients who underwent thalamic DBS (Fields et al., 2003), a handful of studies took a closer look at language outcomes. Wang et al. (2021) analyzed language-related outcomes in relationship to stimulation side and location pre- and post-DBS and found that changes in verbal abstraction had a significant correlation with stimulation location along the anterior–posterior axis within the left VIM. Patients with left ventral anterior-ventral lateral anterior (VA-VLa) nucleus activation performed worse after surgery, whereas those without the left VA-VLa activation showed significantly better performance after surgery, without medication changes pre- and post-DBS in this study. In the only prospective double-blinded randomized trial found in this review, high frequency (120–150 Hz) thalamic VLp stimulation, or areas directly beneath, reduced tremor in patients with ET but worsened verbal fluency (both semantic and phonemic) when compared to low frequency (10 Hz) stimulation and DBS OFF (Pedrosa et al., 2014), while working memory and executive function remained unchanged between groups.

A retrospective study assessed verbal fluency in 13 ET patients with unilateral VIM DBS and 14 PD patients with unilateral STN DBS, in DBS ON and OFF states, compared to 12 HCs (Ehlen et al., 2014). When compared to HCs, patients in both DBS groups uttered fewer words with DBS OFF; however, there were no substantial differences between the DBS cohorts post-DBS. When comparing DBS ON vs. OFF, *post-hoc* analysis revealed that there was a notable reduction in the number of words produced with VIM DBS, particularly in phonemic fluency. Conversely, STN DBS improved phonemic fluency, but this did not suffice to significantly change the overall performance. Decreasing phonemic fluency in patients with VIM DBS was found to be correlated with increasing stimulation amplitudes (Ehlen et al., 2014). Another study by the same group focusing on verbal fluency tasks showed that patients with bilateral VIM DBS produced fewer words than controls, which also worsened with DBS ON state, and was correlated with more anterior electrode positions (Ehlen et al., 2017). VIM DBS can also affect spontaneous language production in ET patients. Ehlen et al. (2016) found that the number of words produced in the verbal fluency tasks was significantly lower in the VIM DBS ON vs. OFF status. A retrospective analysis compared spontaneous language production in a total of 39 participants with VIM DBS for ET, STN DBS for PD, and HCs (Tiedt et al., 2021). Although the study did not show differences in lexical (phonemic) frequency among the three groups, post-hoc analysis showed significantly lower word frequency in the VIM DBS group (with bilateral DBS in 13 out of 14 patients) compared to the STN DBS group while OFF DBS; however, with DBS ON, word frequency improved in the same group. Additionally, both DBS groups showed a lower proportion of open-class words relative to closed-class words when compared to the HC group (Tiedt et al., 2021). To study the effect of VIM DBS on language processing rather than production, Krugel et al. (2014) used an acoustic lexical decision task in a comparative case–control study and found that VIM DBS slowed down word decisions in 10 ET patients and reduced N400 potentials when compared to STN DBS in 14 PD patients and 12 matched HCs.

Three long-term studies followed ET patients for 2 or more years (Heber et al., 2013; Klein et al., 2017; Wang et al., 2020). Among them, the longest study followed 9 ET patients prospectively over 6 years (Heber et al., 2013), who underwent thalamic VL nucleus DBS and were evaluated for cognitive changes before surgery, as well as 1 and 6 years thereafter with DBS ON and OFF. No differences were found in tasks of verbal fluency, memory, executive and intellectual functions comparing pre-surgery, DBS ON, and OFF at 1- and 6-years post-surgery. No medications were changed after surgery. A retrospective review of prospectively collected data, following 9 ET patients with bilateral VIM DBS for up to 2 years (Wang et al., 2020), showed no significant changes in memory, but improvement in anxiety and depression that were seen as early as 1-month post-DBS (DBS OFF) and persisted at 1- and 2-year follow-up evaluation (DBS ON). However, all ET medications were stopped post-DBS, which could possibly affect anxiety and depression. Klein et al. (2017) followed 26 ET patients with bilateral VIM DBS for more than 2 years and analyzed cognitive outcomes pre- and post-DBS relative to their age at surgery. The study found no differences in outcomes between the two groups; however, patients older than 70 years of age had a worse score on the Mattis Dementia Rating Scale preoperatively, which improved post-DBS. Medication changes post-DBS were not reported in this study.

The largest study on cognitive outcomes in ET was a retrospective analysis of 50 ET patients with unilateral (*n* = 37) and bilateral (*n* = 13) VIM DBS (Jones et al., 2020). The study assessed 6 cognitive domains pre- and post-DBS (> = 1 year), while ET medications were continued, and analyzed changes according to baseline characteristics, total electric energy delivery (TEED), and surgery-related complications. Group analysis revealed no significant longitudinal pre- to post-DBS changes for all cognitive domains. *Post-hoc* analysis by age at tremor onset revealed working memory improvement for younger onset ET (<38 years) after DBS surgery, and complications vs. no complications showed a significant decrease in verbal memory in patients with complications after surgery. Additionally, *post-hoc* analysis of cognitive changes by DBS laterality (unilateral vs. bilateral DBS; left vs. right side) did not show any differences in outcomes.

To address the practice effects of repeated exposures to neuropsychological tests, a recent retrospective study utilized regression-based reliable change indices to better objectively assess the impact of DBS on cognition (Kielb et al., 2022). Thirty ET patients with unilateral VIM-DBS underwent neuropsychological evaluation around 6–7 months pre-DBS and 6–7 months post-DBS. Group-level analysis showed no significant changes in cognitive test scores pre- and post-DBS, and individual reliable change (RC) scores showed that 60% of the sample had a stable performance on all tests, and 36.7% had one significant decline in RC score, which represents normal variability. There was no report on whether medications were changed post-DBS in this study.

Looking into cognition-related mood variables, a prospective comparative study of mood, specifically anger, in patients with DBS for PD (STN or GPi) or ET (VIM) showed that STN and GPi DBS were associated with significantly higher anger across pre- to post-DBS as compared to VIM DBS (Burdick et al., 2011). There was no significant change in the levodopa equivalent dose post-DBS placement in PD patients, but whether there were changes in ET medications post-DBS was not reported in this study.

### cZi/PSA DBS

Two studies reported on cognitive outcomes in ET patients post unilateral and bilateral cZi-DBS (Fytagoridis et al., 2013; Philipson et al., 2019). Both studies recruited patients prospectively and followed patients for a year after DBS surgery. Fytagoridis et al. specifically assessed verbal fluency in 17 ET patients with bilateral and unilateral DBS and found that there was a significant decrease in verbal fluency 3 days post-DBS surgery while stimulation was still OFF. This change was not detectable at the group level 1-year post-DBS at both OFF and ON states, hence it is possible that the early decreased fluence could be due to lesioning effect or acute changes postoperatively. It is notable that 4 patients with a 50% reduction in verbal fluency 3 days post-DBS had a sustained reduction of 38% after 1 year. Philipson et al. assessed multiple cognitive domains (memory, executive function, attention, and verbal) in 26 ET patients and found no significant changes 12 months post-DBS at the ON state compared to baseline pre-DBS except for a statistically significant but mild decline in semantic verbal fluency. There were no differences in cognitive measures in patients with bilateral vs. unilateral DBS. Medication changes post-DBS were not reported in this study.

## Discussion

DBS remains a highly effective treatment for pharmacologically refractory ET, and its cognitive safety is of utmost importance to patients. Clinically meaningful cognitive outcomes can be hard to define and study, and long-term follow-up of a large patient cohort with cognitive measures can be challenging. The most recent review on cognitive outcomes in patients with ET who underwent DBS included eight studies (Cernera et al., 2019). By expanding our search to PubMed and Web of Science, and systematically searching the up-to-date literature, we were able to identify a total of 20 studies that met the inclusion criteria up to March 2023. In this systematic review, we detailed these 20 studies that dissected a wide range of cognitive outcomes in ET patients who underwent VIM, VL/VLp, or cZi/PSA DBS, followed over a short and long term.

We found a high degree of heterogeneity in study design, sample size, neuropsychological battery, medication status, and statistical analysis. Only one study was a prospective randomized clinical trial among the 13 prospective studies, and seven were retrospective. Most studies were small, with a median number of 22 ET patients in each study (ranging from 2 to 71). Most studies had short-term follow-up post-DBS, with a median follow of 12 months (ranging from 3 to 70 months). In studies comparing pre- and post-DBS cognitive outcomes, tremor medications were either unchanged although tremor was shown to be improved (Tröster et al., 1999; Fields et al., 2003; Heber et al., 2013; Jones et al., 2020; Wang et al., 2021), changed without a significant effect on cognitive outcomes (Wang et al., 2020; Dhima et al., 2021), changed and controlled for (Woods et al., 2003), or not reported (Burdick et al., 2011; Fytagoridis et al., 2013; Klein et al., 2017; Philipson et al., 2019; Kielb et al., 2022). Statistical analyses and investigating individual-level change over time were highly variable among the reviewed literature. Inconsistent evaluation of change in cognitive function across studies deemed difficult to compare results from one study to another sufficiently. The most robustly studied cognitive outcome was language, specifically verbal fluency speed and other aspects of language functioning. Both VIM and cZi/PSA DBS have been documented to adversely influence verbal fluency and language production. Studies showed that VIM DBS resulted in a decrease in speeded phonemic fluency (Ehlen et al., 2014, 2016, 2017), slowing down in word decision-making (Krugel et al., 2014), and reduced use of open class words (Tiedt et al., 2021). Ehlen and colleagues also documented worse verbal fluency particularly during DBS ON compared to OFF (Ehlen et al., 2016, 2017), with increasing stimulation amplitude (Ehlen et al., 2014) and anterior electrode positions in the VIM (Ehlen et al., 2017).

In cZi/PSA DBS, Fytagoridis et al. (2013) hinted at a possible lesioning effect of DBS on verbal fluency as it decreased 3 days post DBS surgery in the OFF state, which became undetectable on the group level 1 year post-DBS in the ON state, although it continued to be mildly detectable in a small number of patients. Additionally, Philipson et al. (2019) also showed a mild decrease in verbal fluency 12 months post-DBS; however, the only comparison made was to the pre-DBS baseline rather than to post-DBS in the OFF state. Hence, it remains unclear if the decline in verbal fluency is due to a long-lasting lesioning effect vs. stimulation effect. None of the studies evaluated the potential correlation of the number of microelectrode passes with cognitive changes in patients with ET, except one on a correlation of cognitive outcome (anger) with the number of passes of the microelectrode in STN and GPi DBS in patients with PD compared to VIM DBS in patients with ET (Burdick et al., 2011).

With the advent of cZi/PSA DBS for ET patients, comparative studies are necessary to compare cZi/PSA DBS vs. VIM DBS on their effect on cognitive outcomes, especially looking into the effect of DBS parameters on such outcomes, given that cZi/PSA DBS is proposed to target a smaller anatomical area effectively and possibly more efficiently treating tremor (Herzog et al., 2007; Barbe et al., 2011, 2018; Sandvik et al., 2012; Xie et al., 2012; Ramirez-Zamora et al., 2016; Blomstedt et al., 2018; Al-Fatly et al., 2019; Dembek et al., 2020).

Multiple studies looked at the effect of laterality on cognitive outcomes. Most analyses were *post-hoc.* Put together, left-sided VIM stimulation could affect different cognitive domains including verbal abstraction, word recall, and verbal memory performance (Loher et al., 2003; Woods et al., 2003; Dhima et al., 2021). A longer or larger left-sided pulse width (>120 μs) was correlated with overall cognitive decline in one study (Woods et al., 2003) and verbal memory decline in another (Dhima et al., 2021). It was postulated that longer or larger pulse width may activate larger-diameter myelinated axons which could disrupt frontal projections within the cerebello-thalamo-cortical network, potentially affecting verbal memory (Lenka et al., 2017; Dhima et al., 2021), although it is also possible that the increased TEED as a result of larger pulse width could also stimulate the adjacent unwanted fiber causing cognitive side effects. Additionally, left-sided VIM DBS for tremors caused by ET, PD, or MS was associated with worse word recall across all three diseases in a small study (Loher et al., 2003). On the other hand, two studies did not find any significant differences in cognitive outcomes pre- and post-DBS when analyzed by DBS laterality (unilateral vs. bilateral DBS; left vs. right side) (Philipson et al., 2019; Jones et al., 2020).

Based on this review of literature, substantial cognitive decline after VIM or cZi/PSA DBS in ET patients appears to be rare, suggesting that both procedures are generally safe from a cognitive standpoint, especially after taking into consideration their overall benefits on patients’ quality of life (Tröster et al., 1999; Fields et al., 2003; Nazzaro et al., 2012). While overall safe, there are conflicting results regarding the impact on verbal fluency and other aspects of language function. Most studies also have a small sample size, limiting the statistical power of the results obtained, with only one study of a randomized trial. In addition, cognitive changes induced by medication are largely neglected in the literature. There is no standard requirement on the medication changes after VIM DBS for ET, although the commonly used medications for ET, such as propranolol, primidone, and topiramate, could have sedative side effects on patients, which could possibly have beneficial effects on cognitive function if they are reduced or stopped after the DBS surgery. As such, future studies that parametrically manipulate the location and laterality of the active contact and stimulation parameters might be necessary to test specific hypothesis pertaining to the effect of stimulation on specific cognitive outcomes. Additionally, long-term prospective blinded randomized controlled trials should be designed, considering the medication changes post-DBS and DBS ON/OFF status as a potential confounder for cognitive outcomes. Statistical analysis that considers practice effects and effect size is also warranted to objectively ascertain true impact. Standardization of test battery will also allow a better understanding of the impact on specific cognitive domains.

## Author contributions

JA: Data curation, Formal analysis, Investigation, Writing – original draft, Writing – review & editing. ML: Formal analysis, Investigation, Writing – review & editing. MP: Investigation, Writing – review & editing. WA: Investigation, Writing – review & editing. HH: Investigation, Writing – review & editing. PW: Investigation, Writing – review & editing. TX: Conceptualization, Data curation, Formal analysis, Investigation, Methodology, Supervision, Writing – original draft, Writing – review & editing.
