# Supplementary material for: Cognitive outcomes in patients with essential tremor treated with deep brain stimulation: a systematic review
Source: Front Hum Neurosci. 2024 Feb 2;18:1319520. doi: 10.3389/fnhum.2024.1319520 (PMC10869505; doi:10.3389/fnhum.2024.1319520)
Supplement: Supplementary file 1 [file Table_1.docx]

**Table S1: a detailed summary of 20 articles studying the effect of DBS on cognitive outcomes in patients with Essential Tremor.**

| Refs | Disease (ET, PD), Targets (VIM or PSA or cZi vs STN or GPi),  Side (Unil vs. Bil), Size (n) | Age at Study (yo), Disease Duration (yrs) (Mean ± SD, unless noted) | Design (including DBS duration and/or follow up duration) (mons) | DBS Settings | Neuro-psychological Tests | Outcomes in Cognition (Pre-Post-DBS, ON/OFF DBS, and medication status) |
| --- | --- | --- | --- | --- | --- | --- |
| Kielb 2022 | ET, VIM, Unil (n= 30) | Age at pre-DBS evaluation: 70.4 ± 6.3  Disease duration: not reported  Age at DBS placement: 71.3 ± 6.4 | Retrospective. Neuropsychological (NP) pre- and post-DBS placement. NP evaluation 6.5 ± 3.6 mons before surgery and 6.6 ± 1.9 mons after DBS placed. Paired t-tests and regression-based Reliable Chang (RC) to identify differences in NP test scores in a single individual. A decline on one RC measure can represent normal variability; change across two or more RC measures is considered abnormal. The False Discovery Rate method was applied to account for multiple comparisons. | Not reported | Working Memory Index  Trail Making Test parts A & B  Boston Naming Test-II (BNT-II)  Verbal Fluency Tests (Phonemic, Semantic)  California Verbal Learning Test-II (CVLT-II) (Total Learning Trials 1-5 and Long Delay Free Recall) | Group level analysis showed no significant differences between pre- and post-DBS cognitive test scores. Right- and left-sided DBS groups were compared separately with similar results.  Individual RC scores showed that 60% of samples had stable performance on all tests, 36.7% had one significant decline in RC value. One patient (3.3%) exhibited decline across multiple domains (not clearly related to DBS).  No report on whether medication was changed after the DBS. |
| Tiedt 2021 | ET, VIM, Unil (n=1), Bil (n=13); PD, STN, Unil/Bil not reported (n=13); healthy control (HC) (n=13) | Age at assessment:  ET: 70.2 ± 9.2;  PD: 67 ± 7.6;  HC: 67.5 ± 8.4.  Disease duration:  ET: 15.4 ± 13.6;  PD: 13.7 ± 4.8  DBS duration:  ET: 3.5 ± 3.2;  PD: 2.9 ± 1.8 | Retrospective comparative analysis of spontaneous language production in ET, PD and HC. Speech samples from interviews with all participants were analyzed OFF- and ON-DBS.  Lexical frequency (word count) was analyzed, in addition to the use of lexical classes by computing a ratio of open/closed class words. | Amp(V):VIM: Lt (3.13 ± 1.48), Rt (3.32 ± 1.46); STN: Lt (2.78 ± 1), Rt (2.52 ±1.14)  Freq (Hz): VIM: Lt (155 ± 33.1), Rt (152 ± 33.3); STN: Lt (119 ± 23.6), Rt (119 ± 23.6)  PW(µs): VIM: Lt (71.54 ± 15.19), Rt (70 ± 14.77); STN: Lt (64.62 ± 11.27), Rt(64.62 ± 11.27).  TEED (total electric.energy delivered) (µJ): VIM: Lt (273.3 ± 552), Rt (220.1 ± 261.4); STN: Lt (103.1 ± 75.8), Rt (91.5 ± 85.8). | Spontaneous language: lexical frequency and use of lexical classes (i.e., open and closed class words).  Open class words included full verbs, nouns, adjectives, and modal adverbs.  Closed class words included modal and auxiliary verbs, all other types of adverbs, conjunctions, pronouns, particles, prepositions, and articles. | Lexical (phonemic) frequency showed no differences between HC group and either VIM or STN DBS groups.  Post-hoc analysis showed significantly lower word frequency in the VIM group compared to the STN group while OFF-DBS; however, VIM DBS-ON improved lexical frequency when compared to DBS-OFF but did not alter the overall performance relative to other groups.  Both DBS groups showed a lower proportion of open class words relative to closed class words when compared to the HC group.  Medications were not changed between DBS-ON and DBS-OFF sessions. |
| Wang 2021 | ET, VIM, Bil (n=26) and Unil (n=4) | Age at surgery: 67.2 ± 8.9  Disease duration: 28.6 ±17.7  Preop NPT to surgery (mons): 1.8 ±1.3  Postop NPT after surgery (mons): 12.3 ±12 | Retrospective analysis of 30 patients with ET who underwent pre- and post- DBS surgery NP test and language analysis.  Language related outcomes were analyzed in relationship to stimulation side and location. Linear discriminant analysis was further used to validate results.  None of the participants had cognitive impairment on NPT before surgery. | Amp (V): Lt: 2.70 ±1.16; Rt: 2.57 ±0.77  Freq (Hz): Lt: 156.43 ±21.6; Rt: 154.44 ±19.58  PW (µs): Lt: 60 (n = 24), 90 (n = 3), 120 (n = 1); Rt: 60 (n = 22), 70 (n = 2), 80 (n = 2), 90 (n = 1) | General cognition: Mini mental status examination (MMSE).  Learning and memory: List learning, story memory, list recall, list recognition, story recall, and figure recall.  Executive function: Trail making test A and B, stroop color, stroop word, stroop inhibition, stroop switching.  Visuospacial function: figure copy, line orientation, the Wechsler Abbreviated Scale of Intelligence (WASI) matrix reasoning, picture completion.  Attention and information processing: digit span, digit span backwards, digit span sequencing, coding.  Affect: Zung anxiety, apathy scale, Center for Epidemiologic Studies Depression Scale (CES-D).  Language: Picture Naming and Semantic (ie, category) Fluency subtests of the Repeatable Battery for the Assessment of NP Status-A. BNT Controlled Oral Word Association Test (COWAT), which measures phonemic fluency, and Vocabulary and Similarities subtests of WASI-II, which measure expressive vocabulary and verbal abstraction, respectively. | VIM-DBS was not associated with broad changes in cognitive or language function.  Changes in verbal abstraction, measured using the Similarities subtest of WASI-II, had a significant correlation with stimulation location along the anterior-posterior axis within the Lt VIM. Patients with Lt ventral anterior-ventral lateral anterior nucleus (VA-VLa) activation performed worse after surgery whereas patients without left VA-VLa activation showed significantly better performance after surgery.  Outcomes measured with DBS ON.  Medications were not changed after and during the DBS test. |
| Dhima 2021 | ET, VIM, Bil (n= 14), Unil (n=36) | Age at onset: 44.5 (interquartile range, IQR): 23- 56.5.  Age at pre-op NPT: 67, IQR 61-72.  Age at post-op NPT: 7.2 mons (IQR 6.4-9.6).  Disease duration: not reported | Retrospective analysis of cognition and mood pre- and post-DBS surgery.  Group-level change was assessed for tremor, cognitive, and mood scores. Cognitive measures with significant change from pre- to post-op were correlated to treatment, disease, and demographic variables.  Individual-level cognitive decline from pre- to post-op was categorized according to slight (≥ 1 SD) and substantial (≥ 2 SD) decline for each cognitive measure. Further statistical analysis was done for measures where ≥ 10% of the sample declined ≥1 or ≥ 2 SD. These groups differences were examined against treatment, disease, and demographic variables.  Additionally, individual-level psychiatric changes including depressions and anxiety were assessed using rates of improved, unchanged, and worsened symptoms. | Amp (V): Lt: 3 (IQR 2-3.4); Rt 2.8 (IQR 1.9-3.3)  Freq (Hz): Lt: 130-135 (n=37), 160-185 (n=6); Rt: 130 (n=20), 185 (n=1)  PW (µs): Lt: 60 (n=27), 90 (n=16); Rt: 60 (n=60), 90 (n=5). | Processing speed: Symbol Digit Modalities Test (SDMT) oral, Color Word Interference Test (CWIT) word.  Attention and working memory: Wechsler Memory Scale (WMS-III) digit span, WMS-III Letter-Number Sequencing (LNS).  Executive function: CWIT inhibition, CWIT inhibition-switching, Wisconsin Card Sorting (WCST) categories, WCST perseverative errors.  Language: BNT, semantic fluency, phonemic fluency.  Visuospatial: Benton Judgement of Line Orientation.  Verbal memory: Rey Auditory-Verbal Learning Test (RAVLT) learning, RAVLT list B, RAVLT delayed recall, WMS-III logical memory I, WMS-III logical memory II.  Mood: Beck’s Depression Inventory II (BDI-II), Beck Anxiety Inventory (BAI), BAI-A | Group-level cognitive scores remained stable except for improvement in BNT. Group level BAI scores improved from mild to minimal levels, however no change was seen when accounted for tremor-related items.  Individual-level analysis showed that 46% of patients experienced subtle decline in overall cognition, which correlated with higher right-sided stimulation amplitude, as did worsened visuospatial judgment.  Verbal memory performance, in the presence of proactive interference, showed the highest rates of cognitive decline. This decline was associated with larger left-sided pulse width. Increased perseveration during novel problem-solving was associated with higher left-sided stimulation frequency.  Patients with intracranial surgical complications demonstrated higher rates of decline in working memory and visual angle estimation.  Medications reduced in 52% of patients after DBS. Limited *post-hoc* analysis did not show cognitive benefit from ET medication reduction. |
| Jones 2020 | ET, VIM, Bil (n= 13), Unil (Lt: n= 30, Rt: n=7) | Age at DBS: 68.2 ± 10.1.  Duration of disease: 33.1 ±20.4  Age at assessment: not reported | Retrospective analysis of 50 ET patients examining cognitive outcomes in 6 domains at baseline and around 1-year post-DBS in the ON state, and analyzing changes according to baseline characteristics TEED, and surgery related complications.  Included patients with > 30% improvement in tremor after 6 months post-DBS. | Amp (V): 2.7 ± 0.7  Freq (Hz): 154.6 ±23.7  PW (µs): 102.3 ± 34.7  Mean TEED (µJ): 123 ± 20.4 | Executive functioning: Trails Making Test, part B, the color-word trial of the Stroop Color-Word Test (Golden version), and a letter fluency task (COWA).  Working memory: Forward Span and Backward Span trials of the Digit Span subtest from the Wechsler Adult Intelligence Scale-III (WAIS-III).  Language functioning: BNT (total items correctly named) and a semantic fluency test  Visuospatial functioning: Line Test and the Benton Facial Recognition Test  General cognitive function: Dementia Rating Scale-II (DRS-II)  Verbal memory: delayed free recall of the Logical Memory Stories from the Wechsler Memory Scale-III (WMS-III), delayed free recall of the Hopkins Verbal Learning Test-Revised. | Group analysis revealed no significant longitudinal pre-and post-DBS changes for all cognitive domains.  *Post-hoc* analysis of cognitive changes by age at tremor onset revealed working memory improvement for younger onset ET (<38 years) after DBS surgery; by DBS type (Unil vs. Bil), and by side (Lt vs. Rt), did not show any differences in outcomes;  by complications showed significant decrease in verbal memory in patients with complications after surgery.  Subgroup analysis revealed no changes in depression or trait anxiety post-DBS.  ET medications were continued after DBS placement. |
| Wang 2020 | ET,` VIM, Bil (n=9) | Age at DBS: 56.3. SD not reported.  Disease duration before DBS: 11.2. SD not reported | Single-blinded retrospective review of prospectively collected data.  Cognitive function and tremor severity were examined at baseline pre-DBS, 1 mons post-DBS (DSB OFF), 12 mons post-DBS (DBS ON), and 48-mons post-DBS (DBS ON). | Amp (V):  1 mon post-DBS: 1.53 ±0.06;  12 mons follow up: 2.57 ±0.33;  48 mons follow up: 3.22 ±0.35  Freq (Hz): 130  PW (µs): 60 | Full-Scale Intelligence Quotient (FSIQ)  Full-Scale Memory Quotient (FSMQ) Hamilton Depression Scale (HAMD)  Hamilton Anxiety Scale (HAMA) | There were no significant changes in the FSIQ and FSMQ scores in all patients during the follow-up and no  patient-reported changes in memory.  There were significant improvements in patients' depression and anxiety from baseline to the 12 mons and 48 mons follow-ups, however, similar effect was seen at 1 mon follow up when DBS was OFF.  ET medications were discontinued after the DBS surgery in all patients. |
| Philipson2019 | ET, cZi, Bil (n= 6), Unil (n= 20) | Age at DBS surgery: 65.8 ± 6.7.  Disease Duration: 30.6 ± 20.1. | Prospective observational analysis.  NPT was done pre-DBS and 12 mons post-DBS in ON state. | Not reported | ET rating scale (ETRS)  Global cognitive function: Ravens colored matrices, National Adult Reading Test (NART-SWE)  Memory: Claeson-Dahls test, brief visuospatial memory test-revised (BVMT-R), and WAIS-IV.  Executive function: Delis-Kaplan Executive Function System (D-KEFS), and Trail Making Test (TMT).  Verbal fluency: Verbal functioning test (phonemic fluency, semantic fluency, and switching category fluency), Stroop Color Word Interference Test (CWIT).  Attention: Dichotic listening task, Paced Auditory Serial Addition Test (PASAT). | No significant changes in the main cognitive domains (memory, executive function, attention) 12 mons post-DBS compared to baseline except for a statistically significant but mild decline in the semantic verbal fluency.  No differences in cognitive change measures in patients with Bil vs, Unil DBS.  No report on whether medication was changed after DBS placement. |
| Klein 2017 | ET, VIM, Bil (n= 26) | Age at surgery 60 ± 8.1 (group I), 73.5 ± 2.6 (group II).  Disease duration at assessment 32.3 ± 15.7 (group I), 18.4 ± 16.6 (group II) | Prospective observational analysis of cognitive outcomes pre- and post-DBS. Patients were grouped according to age with group I < 70 years (n= 12), group II ≥ 70 years (n= 14).  Mean interval follow up was 27.7 mons (range 12-70) in group I and 23.4 mons (range 3-80) in group II.  Exclusion criteria included cognitive deficits before surgery. | Not reported | MDRS  Frontal Assessment battery (FAB)  Beck’s Depression Inventory II (BDI-II)  Fahn-Tolosa-Marin Tremor Rating Scale. | No significant differences in outcomes were observed between the two groups. Cognitive frontal function (FAB) and depression inventory showed no significant change in between groups and pre- and post-DBS.  Cognitive outcome (MDRS score) was significantly lower in group II-patients preoperatively, which, however, improved post-DBS.  No report on whether medication was changed after the DBS placement. |
| Ehlen 2017 | ET, VIM, Bil (n= 13); HC (n=15) | Age at assessment: HC (69.6 ± 7.6);  ET (69.5 ± 9.4).  Disease duration at assessment: 14.1 ± 9.8  DBS duration at assessment: 2.8 ± 2.3 | Prospective comparative controlled study of ET patients and HC subjects.  Verbal fluency tasks were compared at ON and OFF DBS states. Analysis was done on lexical clusters and switches in verbal fluency performance.  Significant change scores were correlated to stimulation parameters. | Amp (V): Lt: 3.11 ± 1.47; Rt: 3.09 ± 1.32  Freq (Hz): Lt: 146.92 ± 31.66; Rt: 146.92 ± 31.66    PW (µs): Lt and Rt: 60.00 median. | Parkinson Neuro-psychometric Dementia Assessment (PANDA)  German verbal fluency (VF) task, the ‘Regensburger Wortflüssigkeits-Test’, which comprises four conditions as below:  (i) semantic non-alternating (naming vegetables);  (ii) phonemic non-alternating (naming words beginning with ‘s’);  (iii) semantic alternating (naming words for animals and pieces of furniture alternatingly); and  (iv) phonemic alternating (naming words beginning with ‘g’ and ‘r’ alternatingly). | ET patients produced fewer words than HC within lexical clusters. DBS ON compared to OFF aggravated this deficit.  This stimulation effect correlated with more anterior electrode positions  No report on whether medications were changed after DBS placement. |
| Ehlen 2016 | ET, VIM, Bil (n= 12), Uni (n= 1) | Age at assessment: 70.2 ± 9.2  Disease duration assessment: 15.4 ± 13.6  DBS duration assessment: 3.5 ±3.2 | Prospective comparative study assessing spontaneous language production at ON and OFF DBS states. | Amp (V): Rt: 3.32 ± 1.46, Lt 3.11 ± 1.49.  Freq (Hz): Rt: 152.5 ± 33.27, Lt: 162.69 ± 48.63  PW (µs): Rt 60, Lt 60 | Parkinson Neuropsychometric Dementia Assessment (PANDA).  Verbal fluency tasks (semantic non-alternating and phonemic non-alternating)  Neurolinguistic analysis of spontaneous language samples including direct (word class, constituents, morphosyntatic categorizations, word complexity, type of clauses, number of sentences, types of stylistic devices, types of errors, duration of monologue, number of words, number of pauses, pause duration, and articulation ratee), and indirect parameters (speed, type-token-ratio, total pause duration, and tactic sentence structure). | At DBS ON condition, participants used a significantly higher proportion of paratactic as opposed to hypotactic sentence structures.  The mean number of words produced in the VF tasks was significantly lower in the VIM DBS ON vs. OFF condition.  There were no significant ON/OFF difference in the PANDA subtests.  Medications were not changed after DBS placement. |
| Ehlen 2014 | ET, VIM, Uni (n= 13); PD, STN, Uni (n= 14); and HC (n= 12). | Age at assessment: HC (66.2 ±7.2); VIM (69.4 ±9.4); STN (69.4 ±9.4)  Disease duration at assessment:  VIM (15.8 ±13.5); STN (13.8 ±5.0)  DBS duration at assessment: VIM (3.0 ±2.8); STN (3.4 ±2.0) | Retrospective comparative study assessing verbal fluency in ET patients with VIM-DBS and PD patients with STN-DBS, at DBS-ON and OFF, compared to HC. | Amp (V): VIM: Rt: 3.13 ±1.37, Lt: 3.07 ±1.50; STN: Rt: 3.07 ±1.50, Lt: 3.07 ±1.50.  Freq (Hz): VIM: Rt: 148.33 ±32.64, Lt: 151.15 ±32.86; STN: Rt: 120.71 ± 37.51, Lt: 120.71 ±37.51.  PW (µs): VIM: Rt: 70.00 ±14.77, Lt: 71.54 ±15.19; STN: Rt: 69.29 ±20.56, Lt: 73.57 ±21.34. | PANDA  Four Verbal Fluency (VF) conditions tested: semantic non-alternating, phonemic non-alternating, semantic alternating, and phonemic alternating | When compared to HC, both DBS groups uttered fewer words when OFF, however there were no substantial differences between the DBS cohorts post-DBS.  When comparing DBS ON vs. OFF, *post hoc* analysis revealed a notable reduction in the number of words produced with VIM-DBS, particularly in phonemic fluency. Conversely, STN-DBS improved phonemic fluency but this did not suffice to significantly change the overall performance.  Error rates (“wrong category” and “word stem repetition”) were substantially reduced by VIM stimulation.  Decreasing phonemic fluency in VIM-DBS correlated with increasing stimulation amplitudes.  No ON/OFF difference in PANDA.  No change in ET/PD medications after DBS. |
| Pedrosa 2014 | ET, VLp (VIM) or areas directly below, Bil (n=14). | Age at assessment: 62.3 ±14.3  Disease duration at assessment: 25.5 ±13.3  DBS duration: 4.6 ±3.2 | Prospective double-blinded randomized trial comparing the effects of different thalamic-DBS stimulation frequencies (high frequency 120-150Hz, low frequency 10Hz, and OFF), on tremor, cognition, and verbal fluency. | Amp (V): Lt: 2.46 ±.89; Rt: 2.46 ±1.03    Freq (Hz): Bil high at 129 ±8.61 (120-150), low at 10  PW (µs): Lt: 75 ±26, Rt: 76 ±26 | Fahn-Tolosa-Marin-Tremor-Rating-Scale  Verbal fluency: Standardized phonemic and semantic verbal fluency (VF)  Executive function: Stroop-Color-Word-Test (SCW)  Working memory: Digit-span-test (DST) | Compared to low frequency stimulation and DBS-OFF, high frequency stimulation reduced tremor but worsened verbal fluency.  Working memory and executive function remained unchanged between groups.  Low frequency stimulation enhanced verbal fluency although it did not ameliorate tremor.  No report on whether medication was changed after DBS placement. |
| Krugel 2014 | ET, VIM, Bil (n=10); PD, STN, Bil (n = 14); HC (n=12) | Age at the assessment: PD (64.7 ±7.9); ET (68.0 ±10.4); HC (66.0 ±77.3)  Disease duration at the assessment: PD (13.4 ± 5.0); ET (16.0 ±15.8)  DBS duration at assessment: PD (3.4 ±2.6); ET (2.9 ±3.1 | Retrospective comparative study comparing lexical processing in these three groups and DBS-ON and –OFF states. | Amp (V):  VIM: Rt: 3.03 ±1.79, Lt: 2.60 ±1.69.  STN: Rt: 2.86 ±1.34, Lt: 3.01 ±1.24.  Freq (Hz):  VIM: Rt: 146.67 ±29.15, Lt: 150.50 ±30.04.  STN: Rt: 123.57 ± 19.85, Lt: 123.57 ±19.85.  PW (µs):  VIM: Rt: 73.33 ±15.81, Lt: 72.00 ±15.49.  STN: Rt: 66.43 ±12.77, Lt: 68.57 ±14.06. | Acoustic Lexical Decision Task ON and OFF stimulation: processing speed, priming effects, and N400 as NP correlate of lexical stimulus processing. | VIM DBS prolonged (or slowed down) word decisions and reduced N400 potentials.  No comparable ON–OFF effects were present in patients with STN DBS.  No report on whether medication changed after DBS placement. |
| Fytagori-dis 2013 | ET, cZi, Bil (n=2), Unil (n=15) | Age at surgery: 66 (SD not reported)  Disease duration: not reported  DBS duration: not reported. | A prospective study.  Verbal fluency was assessed prior to surgery, and 3 days and 1 year after surgery.  Ten patients were also evaluated by comparing performance ON versus OFF stimulation 1 year after surgery. | Not reported | Verbal fluency test: The test consisted of four different subtests. In the first test the patient was asked to mention as many words as possible beginning with the letter A; in the second test, 5-letter words beginning with M; in the third, names of professions beginning with B; in the fourth, 5-letter names of animals beginning with S. | The total verbal fluency score decreased slightly, but significantly, from 22.7 (SD = 10.9) before surgery to 18.1 (SD = 7.5) 3 days after surgery (p = 0.036).  After 1 year, the score was non-significantly decreased to 20.1 (SD = 9.7, p = 0.2678). There was no detectable difference between stimulation ON and OFF after 1 year.  A sustained reduction in verbal fluency was noted in 4 patients who had 50% reduction in verbal fluency 3 days post-DBS, which remained 38% reduced after 1 year.  No report on whether medication was changed after DBS placement. |
| Heber 2013 | ET, VL, at 1year Unil (n=9), at 6 years Unil (n=4), Bil (n=5)  Note:  Electro-des targeted the bottom of the VL or below it. | Age at surgery: 65.6 ±9.1  Disease duration at assessment: 24.0 ±16.2  DBS duration: not reported. | A prospective study of 9 ET patients who were examined before surgery, and at 1 and 6 years thereafter with DBS switched ON and OFF.  Patients were examined 2 weeks (2.2 ±1.5 weeks) before-surgery as well as 1 year (11.2 ± 4.1 mons) and 6 years (70.3 ± 26.8 mons) post-surgery. | Amp (V):  At 1 year: Lt: 1.9 ±0.6 At 6 years: Lt: 2.3 ±0.6); Rt: 2.0 ±0.6)  PW: not reported  Freq: not reported | Verbal fluency:  Vocabulary and Reasoning of the ‘‘Leistungsprufsystem’’ (LPS) .  ‘‘Regensburger Wortflussigkeitstest’’ (RWT) (a test to assess phonemic and semantic word fluency)  The Color–Word-Interference test (a modified Stroop test)  Memory:  Wechsler Memory Scale (WMS)-digit span forward  WAIS-R-digit span backwards (a test assessing verbal working memory and executive function)  Corsi Block Tapping Test (an assessment of nonverbal short-term memory)  German version of the Rey Auditory Verbal Learning Test (VLMT) (to assess auditory–verbal memory).  Reaction time test: The subtest ‘‘Alertness’’ of the Test for Attentional Performance (TAP). | When comparing pre-surgery, DBS-ON, and DBS-OFF at 1- and 6-years post-surgery, there were no differences in tasks of verbal fluency, memory, and executive and intellectual functions.  There was an increase in simple reaction time in the comparison of DBS-OFF 1- and 6-years post-DBS with pre-surgery. DBS-ON improved this reaction time in some patients.  Medications were not changed after DBS. |
| Burdick 2011 | ET, VIM, Unil (n=71); PD, STN, Unil (n= 195); PD, GPi, Unil (n=56). | Duration of disease at surgery: ET VIM (28.5 ± 18.6); PD GPi (12.6 ± 5.1), PD STN (10.8 ± 4.9)  Age at DBS surgery: ET (68.3 ± 11.3); PD GPi (61.1 ± 7.0); PD STN (59.4 ± 9.4) | Prospective comparative study of mood (anger) in patients implanted with Unil DBS for PD (STN or GPi) or ET (VIM).  Pre- and 4-6 mons postoperative Visual Analog Mood Scales (VAMS) scores for all three groups were compared; additionally, pre- and 1-3 mons scores were compared for STN and GPi. | Not reported | Mini-mental status examination (MMSE) score  Visual Analog Mood Scales (VAMS) | STN and GPi DBS for PD were associated with significantly higher anger scores pre- to post-DBS as compared to VIM for ET.  ET patients who on average have much longer disease durations did not get angrier.  No significant change in the levodopa equivalent dose post-DBS placement in PD patients.  Changes to ET medications post-DBS were not reported. |
| Fields 2003 | ET, VIM, Unil (n=40) | Age at assessment: 71.7 ±8.8  Disease duration at assessment: 18.2 ±12.9 | Prospective study. NP assessments were done about 1 mon before and 3 and 12 mons post-DBS.  For group analysis, data were subjected to multivariate analyses, and significant results were further analyzed using univariate techniques. | At 3 mons:  Amp (V): Lt: 3.40 ±0.60; Rt: 3.24 ±0.75  Freq (Hz): Lt: 147.14 ±25.62; Rt 153.00 ±11.51  PW (µs): Lt 89.14 ±23.56; Rt 84.00 ±25.10  At 12 mons:  Amp (V): Lt 3.18 ±0.53; Rt 3.24 ±0.75  Freq (Hz): Lt 150.57 ±25.72;Rt 155.00 ±13.69  PW (µs): Lt 100.29 ±32.49; Rt 84.00 ±25.10 | Cognition: MDRS, deviation score.  Attention: Wechsler Memory Scale-Revised, Brief Test of Attention, Stroop NP Screening Test  Executive function: Wisconsin Card Sorting Test (WCST)  Language: Semantic (category) fluency, Lexical (letter) fluency, Boston Naming Test (BNT)  Visual perception and coordination: Grooved pegboard, Benton Facial Recognition, Hooper Visual Organization Test (HVOT)  Verbal memory: California Verbal Learning Test (CVLT), Wechsler Memory Scale-Revised (WMS-R) | Semantic verbal fluency declined significantly in four (10%) of the patients. who had diminished lexical verbal fluency at baseline.  Significant improvements on cognitive screening measure (MDRS), and on tasks of fine visuomotor coordination, verbal memory and word recognition. The improvements were observed at 3- and 12- months post-DBS as compared to pre-surgery.  No group-wise declines in cognition were observed, but at 12-months post-DBS, more patients showed declines than improvements on language and visual memory tests.  Medication changed after DBS: 19 patients used a beta blocker at baseline, 16 at 3 and 12 mons post-DBS. 19 patients used Primidone at baseline, 15 at 3 mons, and 9 at 12 mons. No analysis on the effect of medications change on cognition. |
| Woods 2003 | ET, VIM, Unil (n=49) | Age the assessment: ET-D (patients with post-surgical cognitive decline): 73.1 (7.2); ET-S (Cognitively stable patients): 70.6 (8.8)  Age at disease onset: ET-D: 42.2 (4.8); ET-S: 35.0 (19.8)  Disease duration: ET-D: 31.1 (14.7); ET-S: 35.6 (18.7)  DBS duration: reported as approximately 3 mons for both groups | Prospective study to identify predictors of cognitive decline after VIM-DBS in patients with ET. NP testing done 3 mons after DBS surgery.    NP declines were  defined as a post-surgical decrease (relative to baseline) of at least one standard deviation on one or more cognitive tests in at least two domains of functioning.  Two groups were identified, ET-D group for patients evidencing post-surgical cognitive decline and ET-S for cognitively stable ET patients | Amp (V): ET-D: 3.18 (0.63); ET-S: 3.51 (0.65)  Freq (Hz): ET-D: 148.15 (27.64), ET-S: 147.95 (23.28)  PW (µs): ET-D: 105.37 (47.17); ET-S: 79.09 (17.43)  Lt VIM: ET-D: 26 (96%); ET-S: 18 (82%);  Rt VIM: ET-D 1 (4%): ET-S: 4 (18%) | General cognitive impairment: Dementia rating scale total  Attention: Brief test of attention, Stroop color-word, Wechsler Memory Scale-Revised (WMS-R) digits backward  Executive function: Wisconsin card sorting test  Language: COWAT, BNT, category fluency  Visuoperception: Hooper visual organization test  Learning and memory: CVLT total trials, CVLT long delay free recall, WMS-R figural memory.  Mood: Beck depression inventory | Twenty-seven patients (55%) demonstrated mild cognitive decrements relative to pre-surgical baseline (ET-D), whereas 22 patients (45%) were classified as NP stable (ET-S) 3 mons after DBS.  At the group level, ET-D participants had significantly higher PW stimulator settings and were more likely to have undergone Lt (dominant hemisphere) DBS than ET-S participants.  A subsequent step-wise discriminant function analysis revealed that disease onset after age 37 years and higher PW settings (120 µs) were the strongest predictors of post-surgical cognitive decline in this sample  Authors reported controlling for medication changes, but such changes were not reported. |
| Loher 2003 | ET, VIM, Unil (n=2); PD, VIM, Unil (n=5); MS, VIM, Unil (n=2) | Age at surgery: 71.4 (range 48–81)  Age at assessment: not reported  Disease duration: not reported | Prospective analysis of NP testing in 9 patients with unilateral VIM-DBS for medically refractory tremors. Outcomes were measured at least 6 mons postoperatively (mean 9 mons).  Testing at DBS-ON and DBS-OFF condition was obtained within a period of 3 to 4 weeks.  No comparison between disease indications was made. | Not reported | General mental impairment: aphasia and agnosia, MMSE, BNT Agnosia Screening Task  Executive Abilities: Stroop Test, Word Fluency  Constructional Abilities: Constructional Praxis  Learning and Memory: Rey Auditory-Verbal Learning Test (RAVLT), Delayed Recall of Constructional Praxis   Processing Speed and Attention: Alertness  Depression: Beck Depression Inventory | VIM DBS-ON led to decrement of word-recall in the short delay free-recall subtest of the RAVLT.  Subgroup analysis indicated that the impairment in word-recall was related to Lt sided thalamic stimulation.  No report on whether medication was changed after DBS. |
| Troster 1999 | ET, VIM, Unil (n=40) | Age at the assessment: 72 ± 8.5.  Age at the diagnosis of ET: 55 ± 13.8. | A prospective study.  NPT on average 1 ± 1.4 mons before and 3 ± 0.7 mons after DBS surgery with DBS ON | Amp (V): 3.4 ±0.6  Freq (Hz): 145.5 ±25.9  PW (μs): 86.3 ±26.5 | Cognitive screening evaluation: MDRS  Attention and executive function: Wechsler Memory Scale–Revised (WMS-R), Stroop NP Screening Test, Wisconsin Card Sorting Test (WCST)  Language: letter fluency, category fluency, BNT  Visuoperceptual and fine motor functions: Hooper Visual Organization Test, facial recognition, Grooved Pegboard Test  Learning and memory: California Verbal Learning Test (CVLT), Wechsler Memory Scale–Revised (WMS-R)  Mood state: Profile of Mood States | Statistically significant but clinically modest gains were observed on tasks of visuoperceptual and constructional ability, visual attention, delayed word list recognition, and prose recall.  Only lexical verbal fluency (letter fluency) declined significantly after surgery. The decline in letter fluency after DBS was probably of modest clinical importance for at least two reasons. First, the patient group reported improved communication on the modified PDQ after thalamic DBS, and second, no patient’s score declined by ≥ 2 SDs.  No report on whether medications was changed after DBS. |
